# Bioinformatic and Genetic Association Analysis of MicroRNA Target Sites in One-Carbon Metabolism Genes

**DOI:** 10.1371/journal.pone.0021851

**Published:** 2011-07-12

**Authors:** Nicole Stone, Faith Pangilinan, Anne M. Molloy, Barry Shane, John M. Scott, Per Magne Ueland, James L. Mills, Peader N. Kirke, Praveen Sethupathy, Lawrence C. Brody

**Affiliations:** 1 Genome Technology Branch, National Human Genome Research Institute, National Institutes of Health, Bethesda, Maryland, United States of America; 2 School of Immunology and Biochemistry, Trinity College, Dublin, Ireland; 3 Department of Nutritional Sciences and Toxicology, University of California, Berkeley, California, United States of America; 4 Section of Pharmacology, Institute of Medicine, University of Bergen and Haukeland University Hospital, Bergen, Norway; 5 Division of Epidemiology, Statistics, and Prevention Research, Eunice Kennedy Shriver National Institute of Child Health and Human Development, National Institutes of Health, Bethesda, Maryland, United States of America; 6 Child Health Epidemiology Unit, Health Research Board of Ireland, Dublin, Ireland; Governmental Technical Research Centre of Finland, Finland

## Abstract

One-carbon metabolism (OCM) is linked to DNA synthesis and methylation, amino acid metabolism and cell proliferation. OCM dysfunction has been associated with increased risk for various diseases, including cancer and neural tube defects. MicroRNAs (miRNAs) are ∼22 nt RNA regulators that have been implicated in a wide array of basic cellular processes, such as differentiation and metabolism. Accordingly, mis-regulation of miRNA expression and/or activity can underlie complex disease etiology. We examined the possibility of OCM regulation by miRNAs. Using computational miRNA target prediction methods and Monte-Carlo based statistical analyses, we identified two candidate miRNA “master regulators” (miR-22 and miR-125) and one candidate pair of “master co-regulators” (miR-344-5p/484 and miR-488) that may influence the expression of a significant number of genes involved in OCM. Interestingly, miR-22 and miR-125 are significantly up-regulated in cells grown under low-folate conditions. In a complementary analysis, we identified 15 single nucleotide polymorphisms (SNPs) that are located within predicted miRNA target sites in OCM genes. We genotyped these 15 SNPs in a population of healthy individuals (age 18–28, n = 2,506) that was previously phenotyped for various serum metabolites related to OCM. Prior to correction for multiple testing, we detected significant associations between TCblR rs9426 and methylmalonic acid (p  =  0.045), total homocysteine levels (tHcy) (p  =  0.033), serum B12 (p < 0.0001), holo transcobalamin (p < 0.0001) and total transcobalamin (p < 0.0001); and between MTHFR rs1537514 and red blood cell folate (p < 0.0001). However, upon further genetic analysis, we determined that in each case, a linked missense SNP is the more likely causative variant. Nonetheless, our Monte-Carlo based *in silico* simulations suggest that miRNAs could play an important role in the regulation of OCM.

## Introduction

One-carbon metabolism (OCM) comprises a set of reactions involving folate coenzymes and is critical for essential processes including DNA methylation, cell proliferation, and the synthesis of nucleic and amino acids. Insufficient folate or vitamin B12 intake, genetic variation [1], or drug interference [2] can disrupt normal OCM function. OCM dysfunction is linked to severe health complications such as cancer [3], [4], anemia [5] and neural tube defects [6]. Folate-mediated OCM also influences levels of the non-protein amino acid, homocysteine. Elevated homocysteine levels have been linked to an increased risk for neural tube defects [7]. Recent studies have revealed widespread changes in gene expression under folate-deficient conditions [8]. However, the underlying molecular mechanisms of these changes are poorly understood.

MicroRNAs (miRNAs) are ∼22 nucleotide non-coding RNAs that regulate eukaryotic gene expression at the post-transcriptional level [9]. Specifically, they associate with the RNA Induced Silencing Complex (RISC) and guide it to target sites within mRNAs. Once bound to mRNA, RISC induces gene repression through a variety of mechanisms, including direct mRNA cleavage and translational inhibition [10], [11]. Most of the ∼950 currently known human miRNAs [12] are predicted to target hundreds of genes, and many genes are targeted by multiple miRNAs [9], [13].

miRNAs have been implicated in a wide array of fundamental biological processes, such as development [14], lipid metabolism [15], response to environmental stress [16] and innate immunity [17]. Accordingly, mis-regulation of miRNA expression and/or activity has been linked to many diseases including various cancers and cardiovascular conditions [18], and is likely to underlie the molecular etiology of many other disorders.

The role of miRNAs in the modulation of folate-mediated OCM has not been extensively investigated. However, initial studies suggest that folate influences miRNA expression; human lymphoblastoid cells grown under folate-deficient conditions exhibit significant changes in the levels of 24 miRNAs [19]. Given this observation, we examined the possibility of miRNA-mediated regulation of the OCM pathway. Specifically, we applied a computational strategy to predict whether any known human miRNAs are candidate “master regulators” of the genes most commonly associated with OCM. In a complementary analysis, we also assessed whether genetic variants within predicted miRNA target sites in OCM genes are associated with relevant metabolites.

## Methods

### miRNA target predictions

We used the TargetScanS 5.1 algorithm [20] for genome-wide miRNA target prediction. Target predictions were filtered further based on conservation of the miRNA and the 6–8 nt target site. For our primary analyses, we defined conservation as precisely shared nucleotide content between human and two of three other mammalian species (rat, mouse and dog).

### Discovery of candidate master miRNA regulators of OCM genes

We implemented a Monte-Carlo algorithm to identify miRNAs that are predicted to target 42 OCM genes significantly more than expected by chance. First, we randomly selected 42 genes from the human genome under the condition that they have at least one predicted miRNA target site. Then we calculated the number of genes from this randomly selected set that each known human miRNA is predicted to target. By repeating this simulation 10,000 times, we generated a background distribution of the number of predicted target genes for each miRNA, which we then used to calculate an empirical p-value for the number of predicted OCM target genes. We increased the number of simulations (up to 10,000,000) in specific instances in order to obtain a non-zero empirical p-value. To account for differences in the average 3′ UTR length between OCM genes and the randomly selected genes in each simulation, we normalized the number of predicted target genes to the average 3′ UTR length.

### The Trinity Student Study (TSS) Cohort

A population of 2,506 healthy, ethnically Irish individuals consisting of students attending University of Dublin, Trinity College and aged between 18 and 28 years old was recruited over a period of one academic year (TSS cohort). Each participant completed a questionnaire regarding intake of relevant supplements and fortified foods, and gave 30 mL of blood. Genomic DNA was extracted from all samples using a Qiagen QIAamp DNA Blood Mini Kit or a Qiagen DNeasy Kit (Qiagen, UK). Serum folate, red cell folate and vitamin B12 were measured by microbiological assays as previously described [21], [22]. Holotranscobalamin II (holoTC) was directly measured with a commercial, monoclonal technique (Abbott AxSYM, Microparticle Enzyme Immunoassay); total transcobalamin II (total TC) was measured with the same technique after first saturating the protein with cyanocobalamin. Levels of total homocysteine (tHcy), methionine, and methylmalonic acid (MMA) were measured at Bevital AS (www.bevital.no). Ethical approval was obtained from the Dublin Federated Hospitals Research Ethics Committee, which is affiliated with TCD and reviewed by the Office of Human Subjects Research at the National Institutes of Health. Written informed consent was obtained from the participants before commencing the study.

### SNP selection

To identify single nucleotide polymorphisms (SNPs) that might influence miRNA targeting efficiency, we cross-referenced the locations of all SNPs with a minor allele frequency > 0.05 from HapMap Phase III [23] and dbSNP v131 [24] against the locations of all predicted miRNA target sites in the 3′ UTRs of 42 OCM-related mRNAs. This yielded 21 SNPs; however, due to primer incompatibility with multiplex design or assay failure of the original SNP, proxies (r^2^ > 0.95) were selected for 3 of these SNPs. In addition to these 21 SNPs in miRNA binding sites we also tested two SNPs with previously reported functional relevance: TCblR E88del [25] and MTHFR C677T [1].

### Genotyping

Genotypes for all 21 variants were determined by allele-specific extension product mass, detected via matrix-assisted laser desorption/ionization – time of flight (MALDI-TOF) mass spectrometry after undergoing iPlex assay chemistry (Sequenom, San Diego, CA, USA). Primer sequences and assay conditions are available upon request.

Out of the 21 variants, 19 were genotyped successfully, 17 passed quality control criteria, and 15 were confirmed as polymorphic in our sample set and were tested for metabolite associations. Individual assay quality was assessed based on call rate, re-genotyping concordance and population fit to Hardy-Weinberg equilibrium (HWE). Genotyping call rates (i.e., success rates) averaged 97% for the TSS samples, and were at least 95% for all accepted variants. More than 10% of TSS samples were repeated with ≥98% concordance for all accepted variants. Only one SNP (MTHFR rs1537514) was out of HWE (p<0.01). We repeated the assay for rs1537514 using different extension primers, and also separately genotyped a highly linked SNP (r^2^ = 0.973; MTHFR rs1537516), and found that both were out of HWE (p = 2.9×10^−5^ and p = 1.9×10^−4^, respectively). While genotyping error is generally the standard explanation for a SNP not adhering to HWE, it is unlikely in this case due to the consistency across three separate assays. These three assays displayed similar measures of call rates and re-genotyping concordance; therefore, MTHFR rs1537514 was retained for further analysis.

### Statistical analysis

Analysis of variance (ANOVA) was used as the primary test to evaluate each polymorphism for association with metabolite levels. For each SNP, only metabolites that we predicted may be affected based on the known biological function of the gene harboring the SNP were tested. In cases where one genotype was too rare in our sample set to analyze by ANOVA, the Student's t-test was used to compare the two genotypes present. Although some metabolic datasets were not normally distributed, our large sample sizes allowed the use of parametric tests as a screening tool. All significant associations via parametric tests were recapitulated with non-parametric tests (data not shown).

## Results

We selected 42 genes (Table 1) known to be involved in the OCM pathway to test *in silico* for miRNA targeting. In order to minimize the rate of false positive miRNA target site predictions, we used the TargetScanS 5.1 algorithm [20] which was recently identified as one of the top performing prediction strategies [26], and then further filtered the predictions to ensure that both the miRNA and the predicted target site are conserved between humans and at least two of three additional mammalian species (rat, mouse and dog). The use of a conservation filter focuses the analysis on putative miRNA regulation of OCM genes that may be important enough to have persisted across ∼80 million years of evolution [27].

**Table 1:** Genes involved in one-carbon metabolism (OCM).

Symbol	Function category	HGNC	Reference
AHCY	Methylation cycle – regeneration of homocysteine	343	[38]
ALDH1L1	Distribution of one –carbon units	3978	[39]
AMT	Distribution of one –carbon units -glycine cleavage	473	[40]
ATIC	Purine biosynthesis - DNA synthesis	794	[41]
BHMT	Homocysteine remethylation	1047	[42]
CD320	Vitamin B12 cellular receptor	16692	[43]
CUBN	Vitamin B12 intestinal receptor	2548	[44]
DHFR	Folate homeostasis	2861	[45]
DMGDH	Distribution of one –carbon units	24475	[46]
DNMT1	DNA methylation	2976	[47]
DNMT3A	DNA methylation	2978	[47]
DNMT3B	DNA methylation	2979	[47]
FOLH1	Folate hydrolysis (intestinal)	3788	[48]
FOLR1	Cellular folate uptake	3791	[49]
FPGS	Folate homeostasis	3824	[50]
FTCD	Provision of one-carbon units – histidine catabolism	3974	[51]
GART	Purine biosynthesis - DNA synthesis	4163	[52]
GGH	Folate hydrolysis (lysosomal)	4248	[53]
GIF	B12 absorbtion	4268	[44]
GNMT	Glycine methylation – folate homeostasis	4415	[54]
MAT1A	Synthesis of SAM - methylation	6903	[33]
MAT2A	Synthesis of SAM - methylation	6904	[33]
MAT2B	Synthesis of SAM - methylation,	6905	[33]
MMAB	B12 metabolism	19331	[55]
MTFMT	Mitochondrial protein synthesis –formyl-methionyl transfer	29666	[56]
MTHFD1	Distribution of one –carbon units	7432	[34]
MTHFD1L	Distribution of one –carbon units	21055	[35]
MTHFD2	Distribution of one –carbon units	7434	[57]
MTHFD2L	Distribution of one –carbon units	31865	[57]
MTHFR	Distribution of one –carbon units	7436	[1]
MTHFS	Distribution of one –carbon units	7437	[58]
MTR	Homocysteine remethylation	7468	[59]
MTRR	Homocysteine remethylation	7473	[60]
SARDH	Distribution of one –carbon units	10536	[46]
SHMT1	Provision of one –carbon units -cytosol	10850	[61]
SHMT2	Provision of one –carbon units -mitochondrion	10852	[61]
SLC19A1	Reduced folate carrier	10937	[49]
SLC25A32	Mitochondrial folate transporter	29683	[62]
SLC46A1	Proton coupled folate transporter	30521	[49]
TCN1	B12 transport protein	11652	[28]
TCN2	B12 transport protein	11653	[28]
TYMS	Thymidylate biosynthesis – DNA synthesis and repair	12441	[63]

For each gene, the gene symbol, known function, HGNC identification, and literature citations indicating involvement in OCM are provided.

### miR-22 is the top candidate master miRNA regulator of OCM genes

**Figure 1:**
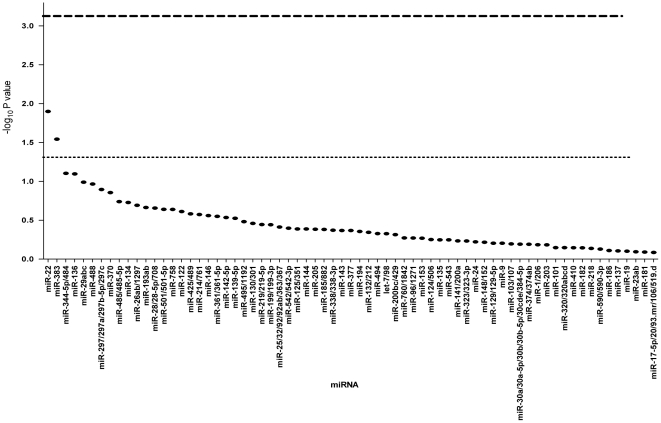
Statistical analysis of predicted microRNA targeting in the one-carbon metabolism (OCM) pathway. For each microRNA, the empirical p-value for the level of enrichment of predicted target sites in OCM genes (Methods) is shown. Analysis is restricted to predicted target sites that, together with their cognate microRNAs, are conserved between humans and at least two other mammalian species among mouse, rat and dog. Lower dashed line indicates p = 0.05; upper dashed line indicates equivalent significance level after correction for multiple hypothesis testing.

Using these target predictions, we implemented a Monte-Carlo algorithm to identify miRNAs that are predicted to target OCM genes significantly more than expected by chance (Methods). The analysis revealed that miR-22 is the top candidate master regulator (p =  0.0126, Figure 1 and Table 2). Though not significant after stringent Bonferroni correction for multiple testing, miR-22 is predicted to have conserved targets in 4 important OCM genes: SLC19A1, MAT2A, MTHFD2 and MTHFR. Notably, the last three genes have at least one miR-22 target site that is conserved among >5 vertebrates, including a predicted target site within MTHFR that is ranked in the 96^th^ percentile according to the TargetScanS scoring criteria. MTHFR also harbors four additional non-conserved miR-22 target sites, indicating a higher likelihood of miR-22 targeting functionality.

**Table 2:** Top 6 candidate microRNA master regulators of one-carbon metabolism genes in mammals.

microRNA	P-value for enrichment of predicted target sites in OCM genes	Predicted OCM target genes
miR-22	0.0126	MTHFD2, MTHFR, SLC19A1, MAT2A
miR-383	0.0286	SHMT2, DNMT3A
miR-344-5p/484	0.0787	DNMT3A, MAT2A
miR-136	0.08	MAT2A, MTHFS
miR-29abc	0.1024	DNMT3A, MAT2A, DNMT3B, MAT1A
miR-488	0.1081	DNMT3A, MAT2A

For each microRNA, the empirical p-value for the level of enrichment of predicted target sites in OCM genes (Methods) and the gene symbol for each predicted target gene are provided. Analysis is restricted to predicted target sites that, together with their cognate microRNAs, are conserved between humans and at least two other mammalian species among mouse, rat and dog.

We repeated the analysis with a less stringent conservation requirement (among primates; human, rhesus, chimp) in order to investigate whether any potential master regulators have arisen recently in order to adapt to evolving physiology. This approach predicts miR-22 target sites within two additional OCM genes, TCblR and TCN2, which are involved in vitamin B12 uptake [28]. However, because the liberal criteria leads to the prediction of many more miR-22 target sites genome-wide, a large proportion of which are likely false positives, the significance of miR-22 targeting of OCM genes is diluted (p = 0.1235). Nonetheless, this less stringent analysis does indeed identify a different candidate master regulator of OCM, miR-125/351, that may have functional relevance in relatively recent evolutionary history (p  =  0.0033; Table S1).

### miR-344-5p/484 and miR-488 are the top candidate master co-regulators of OCM genes

miRNAs often co-regulate their target genes in order to induce effective repression [13]. Accordingly, we implemented a Monte-Carlo algorithm to identify *pairs* of miRNAs that are predicted to co-target OCM genes significantly more than expected by chance. miR-22 does not appear in the top 30 pairs of master co-regulator candidates, suggesting that its influence upon OCM genes may have evolved independently from that of other miRNAs. However, we did identify miR-344-5p/484 and miR-488 as a candidate pair for master co-regulators (p  =  0.0004). Interestingly, though both of these miRNAs are among the top 6 candidates for individual candidate master regulators, neither is statistically significant by itself (Table 2, Figure 1). That their targeting of OCM genes is significant only when considered together as a pair suggests that their influence upon OCM may have co-evolved.

### Genotype-phenotype correlation analysis for polymorphisms within predicted miRNA target sites in OCM genes

In order to investigate the potential phenotypic relevance of the predicted miRNA targeting of OCM genes, we first identified all common HapMap Phase III and dbSNP v131 single nucleotide polymorphisms (SNPs with minor allele frequency > 5%, n = 21) that map to within the predicted miRNA target sites. DNA samples obtained from healthy Irish college students (n = 2,506) were genotyped at each SNP locus. Of these 21 SNPs, 14 were successfully genotyped, and proxy SNPs (r^2^ > 0.95) were genotyped for 3 others. 15 of these 17 SNPs were polymorphic in our sample set (Table S2). Blood, plasma and serum samples from these same students were previously phenotyped for serum metabolites related to OCM. We found significant associations between 2 SNPs and several related metabolites: TCblR rs9426 with serum B12 (sB12) (p < 0.0001), holo transcobalamin (holoTC) (p < 0.0001), total transcobalamin (total TC) (p < 0.0001), total homocysteine (tHcy) (p  =  0.033) and methylmalonic acid (MMA) (p  =  0.045); and MTHFR rs1537514 with red blood cell folate (RCF) (p < 0.0001) (Table 3; Table S2). Upon re-analysis with only those participants who do not take vitamin supplements (which can affect metabolite levels and may make a genetic effect more difficult to detect), all associations remained significant. After Bonferroni correction for multiple testing, the TCblR rs9426 association with sB12, holoTC and total TC and the MTHFR rs1537514 association with RCF remained significant (Table 3).

**Table 3:** Summary metabolite data for individuals with different genotypes at significantly associated single nucleotide polymorphic (SNP) loci that are within predicted microRNA target sites.

SNP	Gene	Predicted microRNA binding site	Genotype	N	tHcy (uM)	Met A (uM)	SB12 (pM)	Sfol (nM)	RCF (nM)	holoTC (pM)	total TC (pM)	MMA A (uM)
rs9426	TCblR	miR-136	CC	2192–2246	*8.7±3.0	N/A	**328±140	N/A	N/A	**57±27	**832±163	*0.188±0.0853
			CT	198–202	*8.2±2.5	N/A	**382±190	N/A	N/A	**88±52	**975±211	*0.176±0.0721
			TT	1	9.2±0	N/A	538±0	N/A	N/A	173±0	1307±0	0.140±0
rs1537514	MTHFR	miR-596 and miR-518a-5p/527	GG	1926–1946	8.7±3.1	29.6±8.5	N/A	34±18	**1057±422	N/A	N/A	N/A
			GC	410–414	8.4±2.5	28.6±7.6	N/A	35±29	**1142±470	N/A	N/A	N/A
			CC	51	8.1±2.3	28.2±7.2	N/A	37±23	**1269±499	N/A	N/A	N/A

Numerical data represent mean metabolite levels ±SD; N indicates the range in the number of individuals with a particular genotype for which data was available for different metabolites. * indicates a significant (p < 0.05) result; ** indicates a significant result (p < 0.05) after correction for multiple testing; N/A indicates that the SNP was not test for association with a particular metabolite.

TCblR rs9426 is in high D′ with another variant within the TCblR gene, E88del (D′  =  0.94; r^2^  =  0.37), which was previously associated with increased levels of MMA in newborns of European ancestry [25] and increased risk of neural tube defects (NTD) in a large Irish population [29]. In order to compare the effects of the two variants, we additionally genotyped E88del in our cohort. Similar to rs9426, E88del displayed significant associations with several metabolites: sB12 (p < 0.0001), holoTC (p < 0.0001), total TC (p < 0.0001), tHcy (p < 0.011), and MMA (p  =  0.0016). However, the differences between alleles in the average level of each of the 5 metabolites are greater for the E88del locus than for rs9426. This observation supports the hypothesis that the deletion of a glutamic acid residue at position 88 (E88del), rather than the removal of possible miRNA regulation (rs9426), is the likely source of the metabolic variation observed between genotypes.

MTHFR rs1537514 is in high D′ (D′ = 0.988; r^2^  = 0.059) with the well-studied MTHFR 677C->T (rs1801133; MAF = 0.24) [1]. Both of these SNPs have minor allele frequencies in our cohort of at least 0.11. We compared phenotypic variation of homozygotes at each SNP locus independently. Specifically, as all MTHFR C667T (SNP1) T (i.e. minor allele) alleles occur on a rs1537514 (SNP2) G (i.e. major allele) background, we compared the following three genotype combinations by a two-tailed t-test: (#1) SNP1 TT, SNP2 GG (n  =  267); (#2) SNP1 CC, SNP2 GG (n  =  728); and (#3) SNP1 CC, SNP2 CC (n  =  49). The results of this analysis reveal that the major (#2) and minor allele (#1) homozygotes of 677C->T differ substantially more from one another in RCF (204 nM difference between means, p < 0.0001) than the major (#2) and minor (#3) allele homozygotes of rs1537514 (152 nM difference between means, p  =  0.0352). This suggests that 677C->T is more likely to be the causative variant.

## Discussion

In this study we describe a computational strategy for the identification of candidate master miRNA regulators of a group of 42 OCM-related genes. Based on our target prediction analyses with the most stringent conservation criteria, we discover a novel role for miR-22 as a candidate master regulator of OCM. miR-22 is widely expressed and has been previously linked to cancer [30], [31]. It is predicted to target OCM genes (MTHFR, TCblR,TCN2, SLC19A1, MAT2A and MTHFD2) that are critical for folate and vitamin B12 transport, folate cofactor distribution and methylation (Table 1). The enzyme encoded by the *MTHFR* gene plays a key role in the regulation of OCM by channeling one-carbon units away from DNA synthesis and into the production of methionine by MTR [32]. Methionine is then converted to the methyl donor, S-Adenosyl methionine (SAM), by MAT2A [33]. Vitamin B12 transport via TCblR and TCN2 influences SAM production by regulating the activity of the B12-dependent MTR enzyme. Folate is required for this process and is transported from the circulation into cells by the reduced folate carrier, the product of the *SLC19A1* gene. Finally, MTHFD2 is important for the exchange of one-carbon units between the cytoplasm and the mitochondrion [34]–[35]. Coordinated regulation of these genes by miR-22 is likely to influence OCM and downstream epigenetic processes.

Our results revealed another potential master regulator of OCM, miR-125/351. The large majority of its predicted targets are conserved only in primates, indicating that its putative role in regulating OCM may be more evolutionarily recent. It also appears likely that while miR-125/351 functions cooperatively with other miRNAs to impose regulation on OCM genes, miR-22′s influence in OCM may have evolved independently from other miRNAs. Notably, both miR-22 and miR-125/351 significantly increase in expression upon folate deficiency [19], lending further support to the prediction that these two miRNAs are relevant to folate-mediated OCM. Our results suggest that miR-344-5p/484 and miR-488 may act in a cooperative fashion to regulate OCM. Neither miR-344-5p/484 nor miR-488 was included in the Marsit et al. study that examined miRNA expression under folate-deficient conditions. Future investigations, including loss-of-function experiments using antagomirs [36] or “sponge” constructs [37] are required to validate the predicted role of miR-22, miR-125/351, miR-344-5p/484 and miR-488 as important regulators of OCM genes.

In a complementary, independent experiment, we genotyped 17 SNPs located within predicted miRNA target sites in OCM genes and found significant associations between two of the SNPs and several metabolites. However, further analyses suggested that these associations could be accounted for by nearby functional variants that are in strong linkage disequilibrium with the miRNA target site SNPs. Nonetheless, we believe that our approach, which combines bioinformatic and genetic experiments, provides a useful model for exploring the role of miRNAs in basic physiological processes.

## Supporting Information

Table S1 **Top 5 candidate microRNA master regulators of one-carbon metabolism genes in primates.** For each microRNA, the empirical p-value for the level of enrichment of predicted target sites in OCM genes (Methods) and the gene symbol for each predicted target gene are provided. Analysis is restricted to predicted target sites that, together with their cognate microRNAs, are conserved among humans, rhesus monkey, and chimpanzee.
(DOC)

Table S2 **Complete metabolite association results for single nucleotide polymorphisms (SNPs) within predicted human microRNA target sites in OCM genes.** * indicates a significant (p < 0.05) SNP-metabolite association; ** indicates a significant SNP-metabolite association (p < 0.05) after correction for multiple testing; N/A indicates the SNP was not present within a predicted microRNA target site.
(DOC)
