# Supplementary material for: Bioinformatic and Genetic Association Analysis of MicroRNA Target Sites in One-Carbon Metabolism Genes
Source: PLoS One. 2011 Jul 12;6(7):e21851. doi: 10.1371/journal.pone.0021851 (PMC3134459; doi:10.1371/journal.pone.0021851)
Supplement: Table S1 — Top 5 candidate microRNA master regulators of one-carbon metabolism genes in primates. For each microRNA, the empirical p-value for the level of enrichment of predicted target sites in OCM genes (Methods) and the gene symbol for each predicted target gene are provided. Analysis is restricted to predicted target sites that, together with their cognate microRNAs, are conserved among humans, rhesus monkey, and chimpanzee. (DOC) [file pone.0021851.s001.doc]

| **microRNA** | **P-value for enrichment of predicted target sites in OCM genes** | **Predicted OCM target genes** |
| --- | --- | --- |
| miR-125/351 | 0.0033 | MMAB, MTHFR, SLC19A1, DHFR, SLC46A1, SARDH, MAT2A, DNMT3B, FPGS, MTR, MAT1A |
| miR-127 | 0.0825 | MTHFR |
| miR-186 | 0.0982 | MMAB, MTHFR, BHMT, CUBN, GGH, DHFR, MAT2A, FOLH1, GART, SLC25A32, MTR |
| miR-1224 | 0.1042 | MMAB, SLC19A1, SLC46A1, MTHFD2L, FTCD, MTR |
| miR-22 | 0.1235 | MTHFR, SLC19A1, MAT2A, MTHFD2, DC320, TCN2 |
