# Supplementary material for: Bioinformatic and Genetic Association Analysis of MicroRNA Target Sites in One-Carbon Metabolism Genes
Source: PLoS One. 2011 Jul 12;6(7):e21851. doi: 10.1371/journal.pone.0021851 (PMC3134459; doi:10.1371/journal.pone.0021851)
Supplement: Table S2 — Complete metabolite association results for single nucleotide polymorphisms (SNPs) within predicted human microRNA target sites in OCM genes. * indicates a significant (p < 0.05) SNP-metabolite association; ** indicates a significant SNP-metabolite association (p < 0.05) after correction for multiple testing; N/A indicates the SNP was not present within a predicted microRNA target site. (DOC) [file pone.0021851.s002.doc]

| **SNP** | **Predicted microRNA binding site** | **Metabolites tested for association** |
| --- | --- | --- |
| BHMT_rs585800 | miR-103/107 | failed assay |
| CUBN_rs780635 | miR-297/297a/297b-5p/297c and miR-567 | sB12, holoTC |
| DHFR_rs1650719 | miR-595 | failed assay |
| DHFR_rs1650721 | miR-518d-5p/519b-5p/519c-5p/520c-5p/526a | failed assay |
| MAT1A_rs1832683 | miR-512-5p | Methionine, tHcy |
| MMAB_rs11067233 | miR-603 | sB12, holoTC |
| MMAB_rs2241201 | miR-335/335-5p | sB12, holoTC |
| MMAB_rs35145546 | miR-1202 | sB12, holoTC |
| MTHFR_677C>T | N/A | tHcy**, Methionine, sFolate, RCF** |
| MTHFR_rs1537514 | miR-596 and miR-518a-5p/527 | tHcy*, Methionine, sFolate, RCF** |
| MTHFR_rs34733339 | miR-125/351 | monomorphic |
| MTHFR_rs45625835 | miR-184 | monomorphic |
| MTR_rs2853523 | miR-563 | tHcy, Methionine, sFolate, RCF, sB12 |
| MTR_rs4659745 | miR-941 | tHcy, Methionine, sFolate, RCF, sB12 |
| MTRR_rs12108985 | miR-499-3p | tHcy, Methionine, sFolate, RCF, sB12 |
| MTRR_rs162036 | miR-361/361-5p | tHcy, Methionine, sFolate, RCF, sB12 |
| SARDH_rs129886 | miR-518d-5p/519b-5p/519c-5p/520c-5p/526a | Sarcosine B, Choline, Glycine A |
| SHMT1_rs1979276.2 | miR-1266 and miR-125a-3p | Sarcosine B, Choline, Glycine A, DMG |
| SLC19A1_rs1051296 | miR-595 | sFolate, RCF |
| SLC46A1_rs2239910 | miR-1252 | sFolate, RCF |
| SLC46A1_rs2239911 | miR-608 and miR-1293 and miR-876-3p | sFolate, RCF |
| TCblR_E88del | N/A | sB12**, holoTC**, total TC**, tHcy*, MMA** |
| TCblR_rs9426 | miR-136 | sB12**, holoTC**, total TC**, tHcy*, MMA* |
